# Effect of fibrous diets on chemical composition and odours from pig slurry

**DOI:** 10.5713/ajas.16.0126

**Published:** 2016-09-09

**Authors:** Conference Thando Mpendulo, Vuyisa Andries Hlatini, Cypril Ndumiso Ncobela, Michael Chimonyo

**Affiliations:** 1 Animal and Poultry Science, School of Agricultural, Earth and Environmental Sciences, University of KwaZulu-Natal, P Bag X01 Scottsville 3209, Pietermaritzburg, South Africa

**Keywords:** Dietary Fibre, Slurry, Odours, Pigs

## Abstract

**Objective:**

Incorporating high fibre ingredients into pig diets has the potential to reduce odour emissions from of pigs. The current study was carried out to determine effect of diets containing 0, 80 and 160 g/kg of each of lucerne hay, maize cobs and sunflower husks on the chemical characteristics and odours from pig slurries.

**Methods:**

Twenty eight pigs averaging 18±2.0 kg were kept in individual cages, over four weeks. All pigs were fed *ad libitum*. Faeces and urine were collected, mixed in a 1:2.3 ratio (w/w), stored and fermented for 16 days in a temperature controlled room at 22°C±2.3°C. The slurry was sampled twice (on day 1 and on day 16) of the fermentation period and analysed for pH, chemical oxygen demand (COD), nitrogen and short chain fatty acids (SCFA) concentration, on wet basis. All samples were tested for odour offensiveness using 18 panelists. A scale of 1 to 5 was used to rank the odour severity, (1 = not offensive, 5 = extremely offensive).

**Results:**

Slurry pH and COD varied with fibre source (p<0.05). On day 16, COD for lucerne hay, sunflower husk and maize cobs were 369, 512, and 425 (standard error of the mean = 34.2) mg of oxygen per litre. Total SCFA concentration was higher at day 16 than day 1 (p< 0.05). Odour offensiveness varied with fibre source across both incubation periods (p<0.05). Sunflower husks and lucerne hay-based diets were rated as less offensive (mean rank = 2.2) than maize cob diets (mean rank of 4.3) (p<0.05).

**Conclusion:**

It was concluded that different fibre sources and incubation period influence chemical composition and odour of the slurry. There is, thereby, a need to incorporate locally available fibrous feeds in the diet of pigs because they have an economical and environmental relevance to pig management.

## INTRODUCTION

Modern pig production systems are criticized for their negative impact on the environment due to nitrogenous gas excretions and odor emission from barns [1]. This is due to large quantities of excreta being produced from pig operations, resulting in loss of gaseous emissions such as ammonia, methane and odors [2]. Although nutrient losses are inevitable, nutritional manipulation can reduce the level of ammonia and odor that is lost in pig facilities by shift partitioning of nitrogen excretion from urea and uric acid in the urine to microbial protein in the feces and lowering the pH of feces by including fermentable carbohydrates in the diet [1,3].

Odor production results from the anaerobic breakdown of nutrients, especially proteins in the gut and in slurry [4,5]. Ingredient composition of the diet affects odors from pig slurry. Incorporating dietary fiber influences the chemical composition and reduces odor emissions [1], hence promoting environmentally friendly pig production. Feeding high fibre diets induces fermentation and consequently increases the excretion of short- chain fatty acids in faeces which decreases the pH value in slurries [6]. Dietary fiber also reduces nutrient loss through pig manure by shifting excretion of nitrogen to feces where it is bound to less volatile bacterial nitrogen. Moreover, the excretion of environmental harmful ammonia is mostly through urine from the urea [1].

Characterization of pig excreta, feces and urine from pigs fed diets diluted with various dietary fiber sources such as Lucerne hay (LH), maize cobs (MC), and sunflower husks (SH) have been explored [7]. The influence of such dietary fiber sources on the changes that occur in the chemical composition and odors from slurry over time is poorly documented. Incubating the slurry, where about half of nitrogen excreted through urine and feces is assumed to be emitted during storage and surface application of the manure, requires a clear understanding of the odors and the changes in chemical characteristics that occur in slurry over standard incubation periods. The breakdown of materials present in manure including protein, which produces the odor might take weeks or longer [1].

The LH, MC, and SH are widely available and are abundant in the tropical region. These fibrous sources do not impose adverse effects on the performance of growing pigs fed fiber inclusion levels below 160 g/kg inclusion levels [5,8,9]. Understanding their effects on odor offensiveness assists in reducing pollution from pig enterprises. Therefore, the objective of the study was to determine the effect of incorporating different fiber sources of varying levels to pig diets on the composition and odors emitted from slurry storage over a 16 day incubation period.

## MATERIALS AND METHODS

### Animal care

The care and use of the pigs was performed following the Certificate of Authorization to experiment on Living Animals from UKZN Animal Ethics Committee (Reference Number: 096/11/Animal).

### Study site and ethical consideration

The experiment was conducted at Ukulinga Research farm, Pietermaritzburg, South Africa. The farm is located at 29°40′ S, 30°24′ E with the latitude of about 775 m above sea level. Daily temperatures average 29°C, with variation ranging from 28.2°C to 43°C. Mean annual rainfall is 735 mm, mostly received in summer, with light to moderate frost occurring occasionally in winter [10].

### Pig management, diets and design

Twenty eight health clinically weaner (male, Pig Improvement Company) pigs weighing an average of 18±2.3 kg were used over a 4-week experimental period including 10 day of adaptation period. They were housed in a room with artificial lighting, heating and ventilation systems. The pigs were confined individually in pens measuring 1.5×1.0 m^2^, containing plastic self-feeders trough and a low-pressure nipple drinker (Big Dutchman Lean Machine, Vechta, Lower Saxony, Germany). The ambient temperature and relative humidity were recorded throughout the experiment at 15 minute intervals using a HOBO TE MPERATURE, RH, 1996 ONSET logger (Onset Computer Corporation, Bourne, MA, USA). The house conditions were kept at a temperature of 21.9°C±2.24°C, 45.2% ±6.85% relative humidity and a 12 h dark-12 h artificial light cycle. The feed and water were provided *ad libitum*. The three fiber sources were chosen based on the availability in the tropics, and to dilute the basal diet. The LH, MC, and SH were ground to pass through a 2 mm screen. The fiber sources were then included at different inclusion levels of 0, 80, and 160 g/kg dry matter (DM) in diet of pigs. It was assumed that the incorporation of fiber would influence the physico-chemical characteristics excreta of pigs.

The conventional diet (Express Weaner) was purchased from a local feed manufacturing company (Meadow Feeds Limited, Pietermaritzburg, South Africa) with a low level of dietary fiber of 50 g/kg DM of total dietary fiber. Ingredients used in the formulation of basal diet were; yellow maize (426 g/kg), soya bean (176 g/kg), soybean oil cake (83 g/kg), wheat bran (100 g/kg), whole wheat (100 g/kg), Oil-sunflower (75 g/kg), cape fish (20 g/kg), and additives (20 g/kg). Table 1 shows chemical analysis of diet containing different fiber sources used in the study. The diet was designed to meet all the nutrient requirements of the pigs. Each pig, representing an experimental unit, was used in completely randomized design. All the four randomly selected pigs per treatment were fed into each of the seven diets including a control.

### Excreta collection, storage and analyses

An amount of 250 g of feces was collected from each pig immediately after defecation. Urine collection was done through placing plastic trays underneath each pen to allow collection of urine that would have oozed through a 1 mm. To reduce the volatilization of nitrogenous compounds in the urine collected, 2 mL of 25% sulphuric acid was added to each tray within 5 minutes of collection. Collected feces and urine samples were stored at 4°C, pending analyses.

The excreta from each pig receiving the same treatment was mixed in 3 L buckets to make slurry for all the samples collected. Slurry was made by mixing feces and urine in a ratio of 1:2.3 (w/w basis of feces and urine) according to Canh et al [11]. Amounts of 200 g of feces and 460 g of urine per pig were sampled on day 1 and 16 of the incubation period of slurry. The slurry was left to stand and ferment at room temperature for a 16 d period in their respective buckets. Sampling for chemical analyses was done on day 1 and 16 of the incubation period of the slurry.

### Measurements

The weight of the experimental pigs was recorded weekly. Feed intake was determined weekly to compute the average daily feed intake (ADFI). The pigs were being weighed once a week to estimate average daily gain (ADG), by weighing feed supplied in and feed left each week. Then feed conversion ratio (FCR) was calculated by dividing feed intake by weight gain. The nitrogen content of the slurry was analyzed using the diffusion technique for soil nitrogen fractionation described by Mulvaney et al [12]. The pH of the slurry was measured on a pH meter, calibrated with certified pH 4 and 7 buffer solutions according to procedures by Lynch et al [13]. Concentrations of acetate, propionate, iso-butyrate, butyrate, and valerate were done following procedures described by Otto et al [14]. The chemical oxygen absorbencies were determined according to Quayle et al [15]. Odor offensiveness was determined using 18 panelists from day 1 and day 16 of incubation period at the Discipline of Animal and Poultry Science at the University of KwaZulu-Natal. Hedonic tone was used to evaluate the odor offensiveness, which was defined as the character of odor (unpleasantness or pleasantness) observed above the odor detection threshold, as described by Otto et al [14].

### Statistical analyses

The effect of dietary fibre source and the fibre inclusion level on the chemical composition of the slurry for two incubation periods was analysed using PROC general linear model of SAS [16]. The PDIFF procedure of SAS [16] was used to separate and compare treatment means. Pair-wise comparisons of means were performed using the PDIFF option. Effects could be considered significant when probabilities were below 0.05. The model used was:

$${\text{Y}}_{\text{ijk}}=\mu +{\text{F}}_{\text{i}}+{\text{L}}_{\text{j}}+{\text{P}}_{\text{k}}+{\left(\text{F}\times \text{L}\right)}_{\text{ij}}+{\left(\text{F}\times \text{P}\right)}_{\text{ik}}+{\left(\text{L}\times \text{P}\right)}_{\text{jk}}+{\left(\text{F}\times \text{L}\times \text{P}\right)}_{\text{ijk}}+{\text{E}}_{\text{ijk}}$$

Where,

Y_ijk_ = dependent variable (pH, chemical oxygen demand [COD], nitrogen, acetate, propionate, iso-butyrate, butyrate, valerate and odour),
μ = the overall mean,
F_i_ = the effect of the type of dietary fibre (i = LH, MC, and SH),
L_j_ = inclusion level (j = 0, 80, and 160 g/kg),
P_k_ = incubation period, (k = Day 1 and Day 16),
(F×L)_ij_ = interaction of fibre type and fibre inclusion level,
(F×P)_ik_ = interaction of fibre type and the incubation period,
(L×P)_jk_ = interaction of fibre inclusion level and the incubation period,
(F×L×P)_ijk_ = interaction of fibre type, fibre inclusion level and incubation periods tested, and;
E_ijkl_ = residual error.

Data on odour offensiveness were square-root transformed before analyses. Pearson’s correlation test was run to cater for the relationship that could exist between the chemical composition of the slurry and the odour offensiveness numerical scales obtained by olfactory sensory evaluation.

## RESULTS

### Effect of feeding fibrous diets on average daily feed intake, daily gain, and feed conversion ratio of pigs

Table 2 gives the impact of LH, MC, and SH incorporation on ADFI, ADG, and FCR of pigs. The ADFI decreased linearly with inclusion level of all fibre based diets (p<0.05). There was an effect of SH in ADG (p<0.05). There was a non-significant relationship between inclusion level of LH, MC, and SH. Increasing levels of all fibre diets illustrated a non-significant effect on FCR (p>0.05).

### Effect of fibre source, inclusion level and incubation period on chemical composition of slurry

The levels of significance for the chemical composition of pig slurry fed different fibre sources at varying inclusion levels and incubation periods are portrayed in Table 3. Fibre source affected pH, propionate, iso-butyrate, butyrate (p<0.05), COD and nitrogen content (p<0.01). Their inclusion level, on the other side, had an impact on total short chain fatty acid (TSCFA) concentrations (p<0.05) particularly butyrate (p<0.05) and valerate (p<0.01). The pH, COD, TSCFA concentrations were influenced by period of incubation (p<0.01). An interaction between fibre source and fibre inclusion level on content of nitrogen and pH was observed (p<0.05). Interaction between fibre source and incubation period had effect on the pH, nitrogen content, the TSCFAs (p<0.05), including iso-butyrate and butyrate (p<0.01). Interaction among fibre sources, fibre inclusion level and the incubation period affected valerate (p< 0.05) and butyrate (p<0.01).

The pH of the slurry was different across all fibre sources during day 1 of incubation period (Table 4). The slurry from pigs fed MC and SH, was, however similar on day 16. The COD was also similar across all fibre sources on the first day of incubation (p>0.05). On day 16, the slurry of pigs fed into SH had a high concentration of COD as compared to the slurry of LH. Nitrogen concentration of slurry from LH tended to be significant (p>0.05). The concentration of TSCFAs were high for SH during day 1, but were highest and the same across all fibre sources during day 16 compared to day 1. The similarity of acetate, propionate and valerate concentration were observed across fibre sources (p>0.05). Isobutyrate and butyrate were the same on day 1, but was largest for MC on day 16 (p< 0.05).

### Odor offensiveness

**Figure 1:**
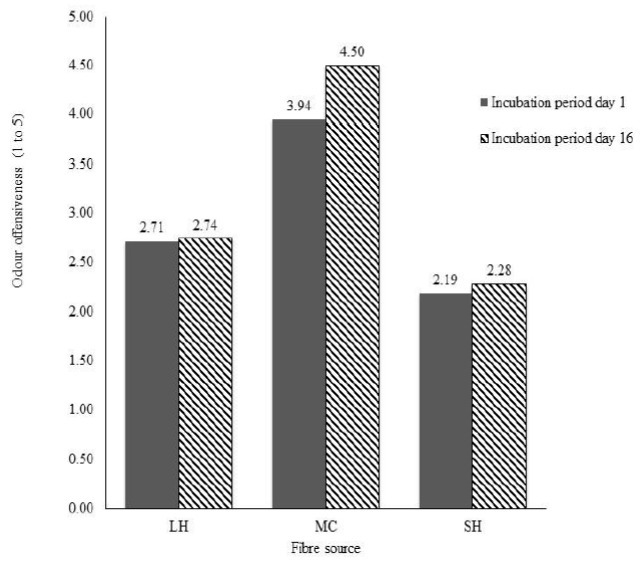
Odour offensiveness from slurry treated with lucerne hay (LH), maize cob (MC), and sunflower husk (SH) over an incubation period of 16 days.

Figure 1, illustrates the effects of dietary fibre inclusion on odour offensiveness. Odour offensiveness of the slurry varied amongst all fibre sources tested (p<0.01). Slurry from pigs fed on diets containing MC had a high odour offensiveness scores than pigs fed into SH. On day 1, the slurry from pigs fed MC was less offensive than the slurry on day 16. The offensiveness of the slurries from pigs fed diets containing LH and SH was similar for both days.

### Correlations

The correlation coefficients are shown in Table 5. There was a positive correlation amongst all chemical parameters tested, except for pH which was negatively correlated to all the chemical parameters tested (p<0.05).

## DISCUSSION

The differences in physico-chemical properties and extent of lignification lead to fibres to be utilised differently in the gut of growing pigs and have variable effects on feed intake and performance [17,8]. Microbial fiber fermentation in the large intestine results in acidification of digesta due to SCFA production, resulting in the reduction of the pH of faeces and manure [18,2,1]. The decrease in pH is partly attributed to the decrease in the ammonium content of the slurry and increased SCFA concentration of the slurry following high fibre fermentation [11]. Likewise, a decline in slurry pH from chickens fed on diets containing molasses when the slurry was incubated for 14 days was reported [19]. In a separate study, it has been highlighted a reduction of pH of faeces and manure when pigs were fed on high fibre diets [20]. Lowering the pH of faeces and manure reduces ammonia emission since ammonia is soluble under its protonated form (NH_4_^+^) [1]. Low pH give rise to efficient retention of nitrogen in the faeces when urine and faeces combine, minimizing volatilization [20,21].

The COD is a measure of water capacity to absorb oxygen during the decomposition of organic matter and the oxidation of inorganic chemicals such as ammonia and nitrite. The findings that fibre source affected COD is in contrast with Gralapp et al [22] who reported no effect on COD in slurry of pigs fed on distillers dried grains. An increase in the COD content of the slurry with time accelerates the de-nitrification process of animal effluents.

Fibre source did not affect nitrogen content of the slurry in the present study. When high fibre diet is fermented, high SCFA concentrations are produced, which contribute to the decrease of pH content of slurry [11,23]. The pH ranges, though statistically different, but were narrow range on Day 1 (7.9 to 8.7) and Day 16 (7.73 to 8.04). The high buffering capacity of the slurries circumvented acidification during incubation even if an accumulation of SCFA occurred [24]. The higher concentration of TSCFA on Day 1 compared to Day 16 for all fibre sources agree with Vedrenne et al [24] who reported a presence of high levels of TSCFA at the start of the incubation and increased at Day 16. The increment of SCFA concentrations in slurry as the incubation time is prolonged could be attributed to the decrease in pH content of pig slurry, as also reported by Hankins et al [21]. The reduction in the microbial activities over time is due to fewer nutrients available in the slurry and a continuous drop of the pH of the slurry.

As fibre content increased in the diet, the malodour emissions are reduced. This can be attributed to an increase in the change of ammonia to nitrate by chemical oxidation, minimizing nutrient volatilization when pigs are fed on fibrous diets [22,13]. In the current study, the slurries from different fibre sources exhibited different pH values. The different fibre sources also have different physical properties and chemical composition [8]. High fibre diets promote microbial synthesis in the hindgut of pigs that yield high levels of SCFA concentrations. A similar process takes place during incubation. Hankins et al [21] and Ziemer et al [25] also highlighted that pH content of slurry also influences nitrogen, SCFAs and microbe activities in pig effluents.

The slurry offensiveness can be explained by the COD, nitrogen and the SCFAs. The COD of the slurry is an important factor in determining odour assessment from the stored slurry [22]. Low COD values and an increase production of iso-butyrate and butyrate may indicate the increased in odour strength. The highest odour scale that resulted from MC-based diets on Days 1 and 16 reflect that maize cobs is relatively digestible and fermentable and producing high concentration of SCFAs such as iso-butyric and butyric acids. High iso-butyric and butyric acid concentration highlights an increase in the level of malodours produced from the slurry [26]. These SCFA concentrations increase odour strength that the slurry from pigs fed on high fibre diets. These fatty acids are one of the leading contributors to odour formation. Low slurry pH values might also have influenced the balance between the volatile and the non-volatile compounds. Odour is a combination of various unpleasant compounds that from animal effluents such as pig slurry [20,27].

## CONCLUSION

The COD varied greatly with fibre source on Day 16 of incubation period. Sunflower husk had highest COD than other fibre source-based diets on Day 16 of incubation. The pH values were different among the fibre- based diets on Day 1. On Day 16, the pH changed except for sunflower husk. Maize cobs-based diet yielded high concentrations of butyrate and iso-butyrate than other fibre sources. LH-based diet yielded high concentrations of acetate on Day 16 of incubation. Maize cobs-based diet produced more odours as compared to other fibre-based diets. Sunflower husk was the least offensive. Fibre source, therefore, need to be considered as nutritional strategy to reduce gaseous emission and odour of pig slurry.

## Figures and Tables

**Table 1:** Chemical composition of the conventional ration and diluted rations (lucerne hay, maize cob, and sunflower husk) used, as fed basis at different inclusion levels

Composition	Inclusion level (g/kg)	Lucerne hay	Maize cob	Sunflower husk	Control diet
Dry matter (g/kg)	80	989.31	989.32	989.32	989.39
	160	990.07	990.40	989.43	
Gross energy (kJ)	80	18.14	18.03	18.20	18.12
	160	18.00	17.94	18.31	
Crude protein (g/kg)	80	192.64	181.75	184.90	195.73
	160	187.02	168.11	166.70	
Ether extracts (g/kg)	80	49.22	51.25	54.13	52.94
	160	45.27	45.92	55.48	
Ash (g/kg)	80	63.19	59.08	56.62	61.19
	160	65.10	54.91	53.85	
Crude fibre (g/kg)	80	46.08	36.46	55.61	26.15
	160	67.09	47.91	86.34	
Neutral detergent fibre (g/kg)	80	228.13	210.65	243.14	192.38
	160	261.16	234.42	269.82	
Acid detergent fibre (g/kg)	80	101.23	101.57	94.56	88.45
	160	126.02	127.48	110.71	
Nitrogen (g/kg)	80	30.85	29.74	26.76	31.36
	160	29.92	26.90	23.34	

**Table 2:** Effect of fibre source and inclusion level on average daily feed intake (ADFI), average daily gain (ADG) and feed conversion ratio (FCR)

Item	Fibre source	Inclusion (g/kg)			SEM	Significance

		0	80	160		
ADFI (kg/d)						
	LH	3.60	2.92	3.66	0.231	**
	MC	3.58	3.31	2.98	0.231	*
	SH	3.41	3.26	3.43	0.231	*
ADG (kg/d)						
	LH	0.84	1.08	1.14	0.137	NS
	MC	0.91	0.93	0.81	0.137	NS
	SH	0.98	0.92	0.95	0.137	*
FCR						
	LH	0.23	0.39	0.32	0.0808	NS
	MC	0.26	0.30	0.27	0.0808	NS
	SH	0.28	0.30	0.28	0.0808	NS

SEM, standard error of the mean; ADF, average daily gain; MC, maize cob; SH, sunflower hulls; LH, lucerne hay; ADG, average daily gain; FCR, feed conversion ratio.

* p<0.05;

** p<0.01;

NS, not significant (p>0.05).

**Table 3:** Significance levels for fibre source, fibre inclusion level, and the incubation period on pH, chemical oxygen demand, nitrogen and short chain fatty acids

Fixed effects	Parameters								

	pH	COD (mgO_2_/L)	Nitrogen (g/kg)	TSCFAs (g/kg)	Ac (g/kg)	Pr (g/kg)	i-But (g/kg)	n-But (g/kg)	Val (g/kg)
Fibre source	*	**	**	NS	NS	*	*	*	NS
Fibre inclusion level	NS	NS	NS	*	NS	NS	NS	*	**
Incubation period	**	**	NS	**	**	**	**	**	**
Fibre source×fibre inclusion level	**	NS	*	NS	NS	NS	NS	NS	NS
Fibre source×incubation period	*	NS	*	*	NS	NS	**	**	NS
Fibre source×fibre inclusion level×incubation period	NS	NS	NS	NS	NS	NS	NS	**	*

COD, chemical oxygen demand; TSCFAs, total short chain fatty acids; Ac, acetate; Pr, propionate; i-But, iso-butyrate; n-But, butyrate; Val, Valerate.

** Indicates the level of significance at p<0.01;

* indicates the level of significance at p<0.05;

NS, indicates insignificance at p>0.05.

**Table 4:** Least square means (±SE) for the effects of period of incubation and fibre type on the pH, chemical oxygen demand, nitrogen and short chain fatty acid concentrations, on wet basis

Parameter	Day 1			Day 16			SEM	p-value
	
	LH	MC	SH	LH	MC	SH		
pH	7.99^c^	8.77^b^	8.32^e^	7.73^a^	8.03^d^	8.04^de^	0.117	0.0318
Chemical oxygen demand (mg O_2_/L)	366^a^	348^a^	362^a^	369^a^	425^b^	512^c^	21.43	0.0001
Nitrogen (g/kg)	5.03bcd	4.70^ab^	4.90abc	5.60^d^	4.40^a^	5.40^cd^	0.200	0.0551
Total short chain fatty acids (g/kg)	10.3^ab^	9.58^a^	11.3^b^	33.1^c^	33.1^c^	32.0^c^	0.422	0.0117
Acetate (g/kg)	6.80^a^	6.80^a^	7.36^a^	25.9^c^	24.0^b^	23.9^b^	0.585	0.0006
Propionate (g/kg)	1.70^ab^	1.43^a^	2.34^bc^	2.97^cd^	3.37^d^	3.65^d^	0.266	0.3770
Iso-butyrate (g/kg)	0.20^a^	0.16^a^	0.20^a^	0.87^b^	1.22^c^	0.96^b^	0.057	0.0065
Butyrate (g/kg)	1.41^a^	0.98^a^	1.29^a^	2.50^b^	3.53^c^	2.32^b^	0.197	0.0015
Valerate (g/kg)	0.19^a^	0.21^a^	0.15^a^	0.84^b^	1.00^bc^	1.14^c^	0.085	0.1483

SEM, standard error of the mean; LH, lucerne hay; MC, maize cob; SH, sunflower husk.

a,b,c,d,e Within a row, means with the different superscripts differ (p<0.05).

**Table 5:** Pearson’s correlation coefficients among slurry characteristics

Measure	pH	COD	N	Ac	Pr	i-But	n-But	Val	TSCFA
Odour	0.12**	NS	−0.28**	0.09*	NS	0.21**	0.20**	0.09*	0.10**
pH	-	−0.19**	−0.41**	−0.51**	−0.38**	−0.44**	−0.37**	−0.41**	−0.53**
COD	-	-	0.13**	0.51**	0.57**	0.41**	0.15**	0.42**	0.49**
N	-	-	-	0.23**	0.20**	NS	NS	0.16**	0.17**
Ac	-	-	-	-	0.75**	0.91**	0.66**	0.79**	0.99**
Pr	-	-	-	-	-	0.69**	0.53**	0.67**	0.73**
i-But	-	-	-	-	-	-	0.83**	0.77**	0.92**
n-But	-	-	-	-	-	-	-	0.59**	0.70**
Val	-	-	-	-	-	-	-	-	0.81**

COD, chemical oxygen demand; N, nitrogen; Ac, acetate; Pr, propionate; i-But, iso-butyrate; n-But, butyrate; Val, Valerate; TSCFA, total short chain fatty acids; Odour, odour offensiveness.

Significance level:

** p<0.01;

* p<0.05;

NS, not significant (p>0.05).
